# Enhancing Internal Learning in Teams: The Role of Network Centrality and Psychological Capital of Undergraduate Students

**DOI:** 10.3389/fpsyg.2020.02197

**Published:** 2020-09-10

**Authors:** Rosa Lutete Geremias, Miguel Pereira Lopes, André Escórcio Soares

**Affiliations:** ^1^Higher Institute of Social and Political Sciences, University of Lisbon, Lisbon, Portugal; ^2^Centro de Administração e Políticas Públicas, Instituto Superior de Ciências Sociais e Políticas, Universidade de Lisboa, Lisbon, Portugal; ^3^Faculty of Health and Life Sciences, Coventry University, Coventry, United Kingdom; ^4^Faculty of Economic Sciences and Management, Nicolaus Copernicus University in Toruń, Toruń, Poland

**Keywords:** network centrality, psychological capital, self-efficacy, optimism, hope, resilience, internal learning in teams, undergraduate students

## Abstract

This study aims to analyze the mediating role of psychological capital (PsyCap) in the relationship between network centrality and internal learning in teams. A questionnaire was administered to 480 undergraduate students to test this relationship. The results confirmed the positive relationship between network centrality and internal learning in teams, and a mediating role of PsyCap in the relationship between student network centrality and internal learning in teams. This study suggests that it is important to promote centrality in advice networks among undergraduate students. In addition, this study might raise awareness among students, teachers, and public policymakers about the need to promote a socially responsible environment in higher education institutions.

## Introduction

The learning development can be considered complex because it can flow within a community since its members can have similar experiences, share the same language, and have a mutual understanding of specific problems (Huber, 1991). In particular, learning has been positively related to the cognitive and innovative adaptation of individuals, measures of network centrality, and performance improvement in different contexts (Gray and Meister, 2006; Wang et al., 2014; Grando et al., 2018). In educational settings, You (2016) has found that the development of students’ learning skills allows them to overcome uncertainties regarding the labor market, due to high unemployment rates and low job security, and facilitates the achievement of future goals.

Internal learning processes involve the creation of knowledge by individuals, and it is necessary for individuals to realize that it is an issue over which they can exert some influence (Kessler et al., 2000; Goorha and Mohan, 2009). Therefore, understanding the factors that enhance the student learning process is important for uncovering why some students are successful and others are not (Fox and Ronkowski, 1997). Given that previous studies provided evidence that the expected benefits of the learning process include increases in students’ civic responsibility and social justice, as well as the development of interpersonal and social problem-solving skills (McElravy et al., 2017).

While broadly ignored in the learning literature, we argue that the social network theory can be interesting to increase the understanding of the internal learning process. Social network theory emerges from people’s efforts to shape their social relationships (Lee and Kim, 2010). From this perspective, the social network indicates that the individual performance can also be determined by the standard environment or relationship structure where individuals are inserted (Carboni and Ehrlich, 2013). On the other hand, Luthans et al. (2014) pointed out that it is important for individuals to be committed to promoting learning and overcoming barriers to success, so for it, they need to develop certain psychological skills.

Although no internal learning studies have included the social network theory directly, more recent research has shown interest in conducting studies that analyze the relationship between individual’s psychological capabilities and his position on different types of social networks (Grando et al., 2018). However, there have been just a few attempts to run studies that allow us to examine the effects of the relationship between psychological capital (PsyCap) and network centrality in the learning process. Despite that, there is evidence of using social network analysis (SNA) in the classroom context (Soares and Lopes, 2017).

Recent studies by You (2016) and McElravy et al. (2017) noted this deficiency and suggested research on this important relationship. Our logic here is based on research that suggests that an individual’s central position in a social network offers greater access to information and promotes greater confidence to learn (Vardaman et al., 2012). Therefore, this study aims to analyze the mediating role of PsyCap in the relationship between network centrality and internal learning in teams. We argue that the analysis of network centrality can provide a great theoretical and practical contribution to a broader understanding of the influence of PsyCap in the internal learning process.

The remaining of this paper is organized as follows. First, we present a review of the literature on network centrality, internal learning in teams, and PsyCap as well as the outline of the hypotheses of the study. Second, the presentation of the methodological options and procedures and the results are described. Third, we present the discussion of the results and outline of the main implications of this study for theory and practice. The paper ends with some concluding remarks.

## Theory and Hypotheses

### Network Centrality

In the last decade, SNA has experienced a golden age, with significant developments and productive expansion. These factors had a rapid impact on the growth of conceptual and empirical studies in substantial areas (Burt et al., 2013; Eid and Al-Jabri, 2016). On the other hand, recent conceptual, empirical, and technical advances have enabled a growing interest in SNA (Wölfer et al., 2015).

Therefore, SNA is an increasingly interdisciplinary field that focuses on a set of actors and the relationships that link them (Borgatti et al., 2009). The theory of social networks aims to explain the structure of relationships between social entities and how social structure influences other social phenomena, such as psychological well-being, reciprocal support and social capital, knowledge management, and financial risk-adjusted performance (Bae et al., 2018; Rossi et al., 2018).

This approach allows for structuring links between network members through certain interdependencies and assumes that these interdependencies explain something about network members (Wölfer et al., 2015). Therefore, the position that each individual occupies in the social network has been considered crucial for the understanding of different results in various contexts (Fang et al., 2015). According to Lee and Kim (2010), studies on social networks highlight the position that the actors occupy, especially the network centrality.

Network centrality is the structural position most strongly linked to performance-related results (Carboni and Ehrlich, 2013). For Bae et al. (2018), centrality can be defined as the power and influence of an actor within a network, where the actor is defined as an individual that has diverse connections with other people. Centrality indicators using SNA are considered important because they allow us to investigate the degree of network connectivity, as well as identify individuals with more and fewer interactions within the network (Alarcão and Neto, 2016).

Based on the position that each individual occupies in a network, different studies, such as Fang et al. (2015), emphasize the role of centrality in instrumental network (also known as advice networks). Advice networks include interactions related to the performance of a particular work or task. Therefore, centrality in advice networks provides individuals with many connecting opportunities to receive and accumulate knowledge, skills, and information related to task performance (Vardaman et al., 2012).

According to Freeman (1979), there are different dimensions that allow us to analyze network centrality, namely degree centrality (in‐ and out-degree), closeness centrality, and betweenness centrality. This current research used the measure of in-degree centrality to analyze the centrality in advice networks. In-degree centrality is the measure most commonly used to show the position of the actor in a network, which is based on the number of direct links that an actor has with other actors (Badar et al., 2015). Additionally, in-degree centrality is considered as the relative extent to which an individual can be connected to all other individuals in the network, thus allowing to quantify the relative number of relationships of an individual in a given context (Vardaman et al., 2012). Furthermore, in-degree centrality allows comparisons across networks of different sizes and it is the most straightforward method to measure the network centrality of actors (Lee and Kim, 2010; Vardaman et al., 2012; Badar et al., 2016).

### Positive Psychological Capital

Positive PsyCap refers to certain positive psychological resources, such as self-efficacy, optimism, hope, and resilience, and has been widely demonstrated as a higher-order construct (Luthans et al., 2018). According to Luthans and Youssef-Morgan (2017), PsyCap is considered as a relevant construct that reflects the positive psychological state of development of individuals.

For Luthans et al. (2006), the PsyCap consists of four capabilities, including: (1) self-efficacy: refers to individual trust which consists in making the necessary effort to successfully perform challenging tasks; (2) optimism: consists in making positive attributions in the realization of future events; (3) hope: related to the perseverance to achieve goals and when necessary to redirect the paths to achieve success; and (4) resilience: refers to an individual’s ability to recover from adversity, even when faced with problems in order to achieve success.

Despite that self-efficacy, optimism, hope, and resilience are depicted as different conceptual capabilities, certain authors such as Luthans et al. (2007) argued that these constructs share a common variation called PsyCap. PsyCap is widely cited in the literature as a malleable construct more open to development, as opposed to trait-like constructs such as the Big five personality factors (Datu et al., 2016; Chen et al., 2019; Xu et al., 2020).

For Luthans and Youssef-Morgan (2017), these aforementioned psychological capacities were the first to be incorporated into PsyCap because of certain criteria, such as adequacy to theory, measurement, development, and impact on performance. These four psychological capabilities together enable an individual to overcome obstacles and remain motivated to achieve goals and success (Harms et al., 2018; Wang et al., 2019).

The identification of PsyCap as a second-order construct has become increasingly common in many studies, especially in the area of organizational behavior (Luthans et al., 2008). Moreover, the interaction of these psychological capacities creates a synergistic motivational effect (Huang and Luthans, 2014). Thus, theoretical and empirical studies have considered PsyCap as an emerging nuclear construct related to different positive outcomes (Luthans et al., 2010).

In the academic environment, previous studies have shown a positive relationship between PsyCap and life satisfaction (Datu and Valdez, 2019), academic performance (Luthans et al., 2012), positive emotions (Carmona–Halty et al., 2018), motivation, engagement, and achievement (Datu et al., 2018). However, further studies are needed to better understand the relationship between PsyCap and other outcomes, especially with regard to internal learning in teams.

### Internal Learning in Teams

The learning experience can be considered as a positive process involving support, motivational satisfaction, and task development. On the other hand, this experience may shift to a counterproductive trajectory, increasing motivational frustration and disengagement from the learning process (Jang et al., 2016). It is for this reason that learning-related activities are considered critical for achieving positive outcomes in different contexts (Song et al., 2014; Sáiz Manzanares et al., 2017).

Internal learning in teams is considered as an ongoing process that involves planning, monitoring, and evaluating different tasks and allows individuals to adapt flexibly the tasks defined to the progress achieved (Pekrun et al., 2002). According to Ortega-Maldonado and Salanova (2017), internal learning in teams refers to the knowledge acquired by students at the end of a study program. Moreover, Edmondson (1999) conceptualized individual learning as a continuous process of reflection and action, characterized essentially by asking questions, seeking feedback, reflecting on results, and discussing errors or unexpected outcomes of certain actions.

According to Goorha and Mohan (2009), the learning process goes through various stages, namely: (1) collecting concrete experience on a concept, (2) reflection and observation on the main aspects of the concept, (3) abstract conceptualization of the concept using reflections, and (4) application of the concept through experimentation. According to Wang et al. (2014), learning is related to the intentional actions of individuals who seek access to knowledge, experiences, insights, and opinions.

Learning is linked to intrinsic motivation, as genuine student interest in course material will facilitate their academic success. Thus, student academic performance is an important indicator of how well the chosen course matches their interests and skills (Conti, 2000). According to Pekrun (1992), in a results-oriented society, learning should be considered one of the most important parts of the student’s daily life. On the other hand, the attention to students’ who worked in a team’s settings can be beneficial to understand the learning process. Hassanien (2006) argues that working in teams can increase the development of internal learning through discussion, analysis of ideas, and assessment of the other member’s ideas. For Dickerson et al. (2013), working in a team provides great learning opportunities, given that it allows team members to build on the ideas of others and enhance their thinking and understanding.

For Roorda et al. (2011), the study of internal learning in teams is considered important, given that it is a predictor of academic success and future career opportunities for students. Thus, analyzing the factors that drive learning in the academic context has been an important priority for students, university administrators, and policymakers (Martínez et al., 2019).

On the other hand, the tasks that students find in universities serve as preparation for the job market, given that the experimental activities, the workgroup, and structured opportunities to interact with other students of the same university resemble tasks and behaviors of employees in organizational contexts (Datu and Valdez, 2019). Students, as well as employees, need to have motivation and energy to learn and achieve their goals, especially undergraduate students who perform their activities in challenging circumstances, thus, they should value the learning process as they prepare for their careers (You, 2016).

### Network Centrality and Internal Learning

Social network analytics has emerged as a powerful approach to understanding how an individual’s position in a social network provides important new insights into patterns and structures of interaction that would otherwise be difficult to achieve, allowing a wide range of outcomes to be influenced (Russo and Koesten, 2005). In this context, the social network perspective complements the traditional focus on individual attributes and emphasizes the relationship between certain actors to better understand their behaviors within the network (Lee and Kim, 2010).

According to Borgatti and Halgin (2011), network centrality is a valuable source of information and proximity to the largest number of network members, as it allows communication on the network to be done quickly. Thus, central individuals are those who can use a network connectivity framework to obtain and make information available quickly and effectively (Mantzaris et al., 2013; Reychav et al., 2017). For Fang et al. (2015), people who occupy central positions in advice networks benefit from useful knowledge and social support and are likely to accumulate task information, which can boost the learning process.

However, there is little empirical evidence to support the positive relationship between network centrality and internal learning in teams. Previous empirical research, such as the study of Mantzaris et al. (2013), provided a theoretical view that allows us to develop the link between network centrality and different positive outcomes. This evidence leads us to formulate the following hypothesis:

*H1*: network centrality positively influences internal learning in teams.

### Network Centrality and Psychological Capital

PsyCap represents individual motivational propensities that accumulate through positive psychological compositions that can be socially constructed (Chen et al., 2019). Authors such as Russo and Koesten (2005) argued that an individual with a high network centrality is in direct contact with many others in the network, which allows the development of certain psychological capabilities.

Additionally, central individuals in advice networks are considered to be successful. Therefore, they have higher levels of hope in terms of capacity and motivation to create alternative pathways leading to the achievement of academic goals and have high efficacy beliefs in their ability to confidently pursue academic goals, resilience in the face of uncertainties, setbacks, failures, and conflicts and optimism in obtaining positive results in stressful situations (Vardaman et al., 2012; Luthans et al., 2018).

For Lee and Kim (2010), network centrality is a potential measure of influence based on the actors who seek to interact within the social network. Network centrality plays a relevant role, as it is the mechanism that allows individuals to obtain information through contact with others, which can increase the positive psychological capabilities that make up PsyCap, namely self-efficacy, optimism, hope, and resilience (Dawkins et al., 2013).

Moreover, network centrality in advice networks consists of a set of interactions that are predominantly used for social and emotional support (Vardaman et al., 2012). Thus, central individuals in social networks can face greater sacrifices as they are rewarded for their value as a source of advice, information, and knowledge (Vardaman et al., 2015). Given this, we hypothesize that:

*H2*: network centrality positively influences psychological capital.

### Psychological Capital and Internal Learning in Teams

PsyCap has been recognized as a driving force for positive outcomes in the academic field (Ortega-Maldonado and Salanova, 2017). Certain studies refer to the positive relationship between PsyCap and academic performance. For example, You (2016) conducted a study with 490 college students, and the results showed that PsyCap has a positive and significant relationship in strengthening learning.

For Luthans et al. (2014), a proactive approach to the development of psychological resources for students can effectively promote learning and help to overcome barriers to academic success. In this context, the PsyCap of students contributes to improve learning and overcome uncertainties and facilitates future achievement of goals (Ortega-Maldonado and Salanova, 2017).

Datu and Valdez (2019) argue that the study of PsyCap in the academic field may be beneficial for the preparation of university students in creating favorable conditions for the learning process. Therefore, You (2016) suggests that education professionals should recognize students’ PsyCap as a valuable resource for learning and, thus, develop effective strategies for assessing and managing PsyCap in the classroom. Given this kind of previous conclusions, we hypothesize that:

*H3*: psychological capital positively influences internal learning in teams.

### The Mediating Role of Psychological Capital

Previous research has established a positive significant link between PsyCap and success in different academic outcomes (Luthans and Youssef-Morgan, 2017). Newman et al. (2017) argued that the social support from peer relations contributes to reinforcing their psychological capacities. For Luthans et al. (2012), PsyCap is a significant antecedent of positive outcomes in the academic field.

Previous studies have also concluded that support from advice networks is positively associated with individuals’ PsyCap and drives the achievement of positive outcomes in different contexts (Nigah et al., 2012). According to Soares and Lopes (2014), network centrality has a special role, as it is the mechanism that allows individuals to obtain information through contact with others. Thus, Dawkins et al. (2013) argued that interactions with different actors can increase the PsyCap and allow the attainment of different positive outcomes. We, thus, hypothesize that:

*H4*: psychological capital mediates the relationship between network centrality and internal learning in teams.

**Figure 1:**
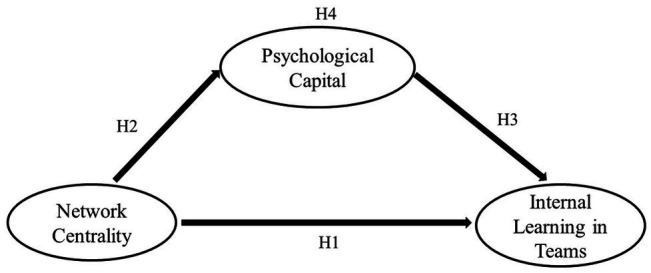
A conceptual model of the relationship between network centrality, psychological capital (PsyCap), and internal learning in teams.

A conceptual model of the mediating role of PsyCap in the relationship between network centrality and internal learning in teams is presented in Figure 1. In addition, the conceptual model presents also the hypotheses under study.

## Materials and Methods

### Participants

Participants in this study were undergraduate students from three large higher education institutions (two public institutions and one private institution). These higher education institutions were selected from a list of eight higher education institutions. As a result, 600 questionnaires were distributed and 480 questionnaires (80% acceptance rate) were received during the month of August 2018 (4 months after the beginning of each module).

In total, 480 questionnaires from 22 classes (ranging from 19 to 44 students per class) were considered valid. Our sample size is greater than the minimum sample size (200) recommended for structural equation modeling analysis with maximum likelihood estimation (Hair et al., 1995). Of the entire sample, the participants were 54% men, and the average age of the participants was 24 years (*SD* = 5.94). The most significant classes were Economics (25%), Business Management (12%), Nursing (11%), and Linguistics-English (8%). Additionally, 61% were from the first year, 21% from the second year, 11% from the third year, and 7% from the fourth year.

### Procedures

The data collection has been authorized by the board of each institution, and permission has been granted by the lecturer of the modules in which the survey took place. Thus, participants voluntarily completed the questionnaire during the class period, using paper and pencil. They first filled out a questionnaire measuring PsyCap and internal learning in teams. Then, a questionnaire measuring network centrality was administered. Moreover, it is important to note that all variables under study were measured at the individual level of analysis. Therefore, for the internal learning in teams variable, we asked the students to fill the questionnaire taking into account their individual work with team member collaboration, as suggested by Lee et al. (2018).

To ensure confidentiality given the sensitive nature of the research, the first author personally distributed and received all questionnaires and also clarified any doubts that arose during this proceeding. Furthermore, all participants were informed that participation was voluntary and the data collected would be processed solely by the researchers involved in this study. Given that we used network centrality measures, we were not able to guarantee the anonymity of the responses, but all the participants were informed of this fact.

### Measures

#### Network Centrality

Network measures were collected by asking participants to nominate up to five same-class colleagues enrolled in a specific module whom they turn to make an important decision related to the school task performance (advice networks). The number of same-class colleagues that each participant indicated is consistent with the research literature (e.g., Lopes, 2012). No list of students was provided in the questionnaire, so participants were free to choose same-class colleagues from their relations. The lack of constraint regarding the minimum number of same-class colleagues that each participant could indicate contributes to minimizing measurement errors, that is, a gap between the actual number of relations and the declared number (Vignery and Laurier, 2020).

Additionally, for each of the nominations, participants rated, “Sufficient” (1) to“Very Much” (7), how much they really turn to the nominees. After the data collection process, we have calculated the degree centrality (in-degree) for each advice networks using UCINET 6.681 software for Windows developed by Borgatti et al. (2002). Given that we used 22 advice networks of different sizes, we follow the recommendations from different authors, such as Vardaman et al. (2012) to correct an individual’s network size by aggregating responses by imputing the size of a network in an individual’s centrality score. According to Freeman (1979), this procedure allows comparing the relative centrality of individuals located within different networks.

#### PsyCap

We used the version of the 24-item questionnaire adapted for academic research by Luthans et al. (2012). The scale is composed by four subscales with six items each, corresponding to positive psychological capacities evaluating, respectively, self-efficacy (e.g., “I feel confident when I look for a solution to a long-term problem”); hope (e.g., “There are lots of ways around any problem concerning my schoolwork”); resilience (e.g., “I usually manage difficulties one way or another concerning my schoolwork”); and optimism (e.g., “In studies, I am optimistic about what will happen in the future”). The responses were given on a six-point Likert scale, from (1) “Totally Disagree” to (6) “Totally Agree”. According to Luthans et al. (2012), the 24-item PsyCap scale presented in the original study has a Cronbach’s αs of 0.90.

#### Internal Learning in Teams

We used the scale developed by Edmondson (1999). The scale was confirmed by Bresman and Zellmer-Bruhn (2013) and is related to internal learning with seven items. Example of items: “We regularly reserve time to find ways to improve the group’s work processes” and “In the team, there is always someone who ensures that we stop to reflect on the work process.” The response scale used is a seven-point Likert type, from (1) “Totally Disagree” to (7) “Totally Agree” with a Cronbach’s alpha of 0.71.

The scales were translated into Portuguese using the translation/retroversion method. The original scale and translated versions were carefully compared, at this stage, an English-speaking native and a Portuguese-English linguistic lecturer assisted us in this process.

### Measure Validity

We ran a confirmatory factor analysis on our two latent constructs: PsyCap and internal learning in teams, omitting network centrality, given that it has single-index scores rather than multi-item. The confirmatory factor analysis, carried out with the AMOS software (V.25) on the PsyCap scale, resulted in adequate values. The model presents moderate and good factorial weights (*λ* ≥ 0.30) and appropriate individual reliabilities (*r*2 ≥ 0.10). The final model has excellent adjustment indexes [*χ*^2^(145) = 242.993, *ρ* < 0.001; TLI = 0.908; CFI = 0.922; GFI = 0.949; SRMR = 0.044; RMSEA = 0.038]. The Cronbach’s alpha for the PsyCap dimension was 0.86. We used PsyCap as a second-order factor, given that PsyCap as a second-order construct has a stronger impact on positive outcomes in the academic field than the four psychological capabilities separately (Alessandri et al., 2018).

For the internal learning in teams scale, the confirmatory factor analysis allowed us to obtain adequate values. The model presents moderate and good factorial weights (*λ* ≥ 0.40) and appropriate individual reliabilities (*r*2 ≥ 0.16). The final model presents excellent adjustment indices [*χ*^2^(7) = 23.797, *ρ* < 0.001; TLI = 0.950; CFI = 0.977; GFI = 0.984; SRMR = 0.034; RMSEA = 0.071]. Cronbach’s alpha coefficient for internal learning in teams is 0.76.

## Results

### Descriptive Statistics

Network centrality data generated with UCINET after correcting participants’ centrality score due to the different sizes of the advice networks were entered into an SPSS V.25 software file along with the other latent constructs, namely PsyCap and internal learning in teams. Table 1 shows the means, standard deviations, Cronbach’s alphas (in parentheses), and Pearson’s correlations among the variables being studied. The internal consistencies obtained for the scales used were good, and the pattern of correlations revealed significant direct relationships for all the variables in our sample.

**Table 1:** Means, standard deviations, and correlations between study variables.

Study variables	*M*	*SD*	1	2	3	4	5	6	7
Network centrality	1.40	0.99	-						
Psychological capital	4.77	0.56	0.124^**^	(0.86)					
Self-efficacy	4.69	0.74	0.051	0.645^**^	(0.70)				
Optimism	5.15	0.81	0.070	0.710^**^	0.252^**^	(0.72)			
Hope	4.83	0.68	0.123^**^	0.720^**^	0.368^**^	0.320^**^	(0.74)		
Resilience	4.41	0.90	0.113^*^	0.781^**^	0.281^**^	0.418^**^	0.449^**^	(0.80)	
Internal learning in teams	3.95	1.19	0.117^*^	0.258^**^	0.196^**^	0.147^**^	0.234^**^	0.175^**^	(0.76)

*N* = 480. Cronbach’s αs (in parentheses).

* The correlation is significant at the 0.05 level (2-tailed);

** The correlation is significant at the 0.01 level (2-tailed).

### Hypotheses Testing

According to Hu and Bentler (1999), the model fit índices suggest an acceptable fit for our hypothesized structural model (*χ* ^2^ = 542.842, *df* = 317, *ρ* < 0.001, TLI = 0.90; CFI = 0.89; GFI = 0.92; SRMR = 0.049; RMSEA = 0.039). Thus, for the hypothesis test, we used a bootstrap approach with a 90% confidence interval over the indirect standardized effects. The results show that network centrality has a positive influence on internal learning in teams (*β* = 0.085; *p* = 0.009) and network centrality positively influences PsyCap (*β* = 0.173; *p* = 0.004). Thus, hypotheses 1 and 2 were supported. PsyCap is significantly and positively related to internal learning in teams (*β* = 0.325; *ρ* < 0.001), which supports H3.

**Figure 2:**
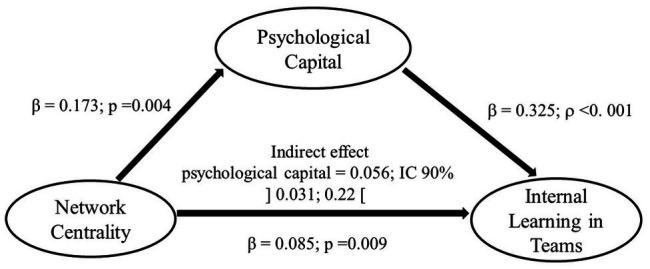
Final model.

The results show that PsyCap fully mediates the relationship between network centrality and internal learning in teams (indirect effect = 0.058; 90% CI limits to 0.030 and 0.104), supporting hypothesis H4. The final model (Figure 2), presents the results of the hypothesis test.

## Discussion

The main purpose of this study was to analyze the mediating role of PsyCap in the relationship between network centrality and internal learning in teams. The results confirmed the positive relationship between network centrality and internal learning in teams (hypothesis 1). These results lead us to argue that academic achievement within the advice networks can contribute to maintaining the link between student centrality and student learning.

According to Kretschmer et al. (2018), undergraduate students tend to cluster with peers who share the same levels of performance. On the other hand, Barnes et al. (2014) argued that this process is unconscious, that is, students during their academic process are probably not opportunistically related to their peers. For Vignery and Laurier (2020), it seems preferable that a student does not resort to other colleagues to address issues related to task performance, than maintaining strategic relationships with underperforming colleagues. So, we can argue that the process of referring colleagues in advice networks can be a strategic choice and is related to learning. Previous research on the centrality of undergraduate students (e.g., Balyer and Gunduz, 2012; Barnes et al., 2014; Kretschmer et al., 2018) has shown positive and significant results between academic achievement and centrality in advice networks.

The relationship between network centrality and PsyCap was also confirmed (hypothesis 2). This relationship seems to happen because, according to Huang and Luthans (2014), PsyCap allows individuals to build confidence and endure hard times in challenging environments. Thus, individuals with high levels of PsyCap are less likely to give up when faced with obstacles and challenges, because they usually have a positive outlook and can look for creative ways to solve problems and, thus, seize opportunities (Chen et al., 2019). For Luthans et al. (2018), PsyCap allows individuals to focus on performing tasks and achieve success in completing these tasks. Therefore, it is understandable that central individuals in advice networks develop positive psychological capacities, such as PsyCap.

For Russo and Koesten (2005), the central position of the individual in social networks might be related to positive behaviors. According to Newman et al. (2014), the study of PsyCap has attracted great interest from academics and professionals and has been related to positive behaviors in different contexts. For example, Newman et al. (2017) argued that access to information may play a critical role in the development of an individual’s psychological resources. This seems to happen because PsyCap represents individual motivational resources that are socially constructed (Dawkins et al., 2013). Thus, we argue that centrality in advice networks can promote the development of students’ PsyCap. Moreover, these results are consistent with previous studies that highlight the benefits of individuals’ centrality in advice networks (Vardaman et al., 2012, 2015).

Our third hypothesis, relating PsyCap and internal learning in teams, was also supported. These results are consistent with previous studies (Luthans et al., 2014; You, 2016), which demonstrated that PsyCap of undergraduate students had a positive and a significant relationship with strengthening learning. Lee and Song (2010) argued that the PsyCap in the academic field contributed to the strengthening of learning outcomes. For Martínez (2019), the development of PsyCap can help students balance the challenges of academic life or at least allow students to evaluate these challenges as manageable, which can facilitate the achievement of high performance. Thus, we argue that PsyCap can also allow students to set goals and work hard to achieve them, which can drive the learning process.

Finally, the mediating role of PsyCap in the relationship between network centrality and internal learning was also supported (hypothesis 4). In particular, these results show that central students in advice networks might develop their psychological capacities in order to achieve learning outcomes in the classroom. These results seem to happen because PsyCap would allow central students to have the confidence to face challenges times throughout their academic careers. For You (2016), PsyCap is a resource that strengthens the learning process, allows students to overcome uncertainties, and facilitates the achievement of future goals.

### Limitations and Future Directions

The present study has some limitations. We excluded the declared nominations corresponding to students who did not respond to the questionnaire related to other variables under study, as recommended by Vignery and Laurier (2020). However, 128 undergraduate students were excluded (average was six students per class), who was nominated at least once and did not answer the entire questionnaire (as they were absent during the data collection process). Thus, we were left without registering these students’ nominations, which could probably increase the centrality scores of the 480 participants. According to Huisman (2009), high levels of non-response in social network studies may underestimate the calculated coefficients. Therefore, future studies should choose to determine network boundaries.

Another limitation relates to a potential common method variance problem, given that the data were collected at the same point in time. However, we argue that same-source bias cannot be considered a threat to the findings of this study, for the following reason. Network centrality data were derived from a sociometric questionnaire, while the remaining variables, PsyCap, and internal learning in teams were measured using a psychometric scale. This is important given that a recommended technique for addressing common method bias is to have predictor and criterion data come from different data sources (Vardaman et al., 2015). For further researches, a longitudinal analysis could help to explore these relations.

Finally, it would be important to use different data collection methods. For Heale and Forbes (2013), the combination of different data collection methods (e.g., qualitative and quantitative) can be important to compare findings from different perspectives. For example, performing interviews of some students who have good network centrality and some who have weak network centrality might add another perspective. Therefore, further studies are necessary in order to explore this alternative path.

### Theoretical and Practical Implications

Our study contributes to the literature in several ways. First, we provide evidence that the centrality in advice networks can influence internal learning in teams, which means that the application of social networking theory in the academic field contributes to achieving learning outcomes. Thus, this study suggests that it is important to promote centrality in advice networks in the academic field. In addition, this study might raise awareness among students, teachers, and public policymakers about the need to promote a socially responsible environment in higher education institutions.

The second contribution of this study comes from explaining the mechanism by which centrality in advice networks can promote internal learning in teams. In hypothesis 4, we proposed PsyCap as the mediator of this relationship. This result is interesting because it reinforces the advantages of PsyCap beyond the organizational context and might contribute to the management of the PsyCap of undergraduate students. For Luthans et al. (2014), the development of PsyCap can be beneficial to promote academic success and, thus, contribute to the preparation of valuable human resources for a professional career.

Our third contribution comes from the notion that centrality in advice networks may be useful in fostering PsyCap. These findings fill an important gap in the PsyCap field, which led certain authors such as Grando et al. (2018) to highlight the importance of explaining the role of an individual’s position on PsyCap fostering (Grando et al., 2018). For Heled et al. (2015), the creation of the dissemination mechanisms that enable individuals to share their knowledge and insights has been theorized as a factor that promotes PsyCap. Given this, PsyCap training programs should have individuals’ centrality in social networks and group work into consideration.

Finally, this study emphasizes the need for effective relationship building within higher education institutions through different interactions between students. So, we argue that this process may contribute to enhance centrality in advice networks. On the other hand, specific efforts should be done to promote student interactions. For example, teachers should focus more on group work and lectures promoted by students as priorities in the teaching and assessment process.

## Conclusion

The main purpose of this study was to analyze the mediating role of PsyCap in the relationship between network centrality and internal learning in teams. Knowing more about the relationship between centrality in advice networks and internal learning of undergraduate students is relevant, given the different positive learning outcomes, such as cognitive and innovative adaptation of individuals, the development of performance in different contexts and engagement (Gray and Meister, 2006; Wang et al., 2014; You, 2016; Grando et al., 2018).

The study results show that network centrality appears to play a positive role in the internal learning in teams of undergraduate students. In addition, the study also found evidence of the relationship between network centrality and PsyCap. Also, as predicted, PsyCap has related to internal learning in teams. Finally, we find evidence of the mediating role of PsyCap in the relationship between network centrality and internal learning in teams. As such, we argue that the present study is important because it suggests specific efforts to promote internal learning in teams in academic settings. In particular, the importance of network centrality and PsyCap should not be underestimated by students, teachers, and public policymakers interested in enhancing internal learning in teams.

## Data Availability Statement

The raw data supporting the conclusions of this article will be made available by the authors, without undue reservation.

## Ethics Statement

The studies involving human participants were reviewed and approved by higher education institutions in the province of Huíla, Angola. It is the only condition required for the data collection process in higher education institutions in Angola. The patients/participants provided their written informed consent to participate in this study.

## Author Contributions

RG designed, prepared, carried out the data collection process, and written the article. ML revised the section of the analysis and discussion and corrected the entire manuscript. AS analyzed and verified the data in this article. All authors contributed to the article and approved the submitted version.

### Conflict of Interest

The authors declare that the research was conducted in the absence of any commercial or financial relationships that could be construed as a potential conflict of interest.
